# Severe Ocular Cowpox in a Human, Finland

**DOI:** 10.3201/eid2112.150621

**Published:** 2015-12

**Authors:** Paula M. Kinnunen, Juha M. Holopainen, Heidi Hemmilä, Heli Piiparinen, Tarja Sironen, Tero Kivelä, Jenni Virtanen, Jukka Niemimaa, Simo Nikkari, Asko Järvinen, Olli Vapalahti

**Affiliations:** University of Helsinki, Helsinki, Finland (P.M. Kinnunen, J.M. Holopainen, H. Piiparinen, T. Sironen, T. Kivelä, J. Virtanen, O. Vapalahti);; Finnish Defence Forces, Helsinki (P.M. Kinnunen, H. Hemmilä, H. Piiparinen, S. Nikkari);; Helsinki University Hospital, Helsinki (J.M. Holopainen, T. Kivelä, A. Järvinen, O. Vapalahti);; Natural Resources Institute Finland (Luke), Vantaa, Finland (J. Niemimaa)

**Keywords:** conjunctivitis, cowpox, cowpox virus, keratitis, orthopoxvirus, PCR, rodent, tecovirimat, vole, zoonoses, viruses, Finland

**To the Editor**: We describe cowpox with corneal involvement in a 31-year-old atopic woman who lived in southern Finland and was unvaccinated for smallpox. In August 2009, she noticed irritation and edema in her right eye and sought care from a local physician; she started topical antimicrobial drug therapy and oral cephalexin 2 days later. Over the following week, fever developed (37.6°C –39.0°C), edema developed on half her face, the eye became increasingly painful, and visual acuity decreased. The conjunctiva was severely chemotic and hyperemic, but the cornea was clear and the other eye unaffected.

Microbiologic samples taken from the eye 11 days after onset showed neither bacteria nor respiratory viruses. Orbital tomography results were normal. The patient was hospitalized, and broad-spectrum intravenous antimicrobial treatment (meropenem, vancomycin, valacyclovir, and fluconazole) was started, combined with topical corticosteroids and antimicrobial drugs. Within 2 weeks, the conjunctiva showed necrosis, and epithelial erosions appeared in the lower cornea, but visual acuity normalized (Technical Appendix Figure, panels A, B).

A strong cytopathic effect was observed in Vero cells infected with conjunctival swab (Technical Appendix Table 1), but the virus was unidentifiable by routine methods. In electron microscopy, cell culture and tear fluid samples contained particles with typical orthopoxvirus (OPV) morphology. PCRs for hemagglutinin (*1*) and 14-kDa genes (*2*) verified OPV infection. Additional PCRs and sequencing confirmed zoonotic cowpox virus (CPXV) with strain designation FIN/K2009. Nucleotide sequences of the hemagglutinin, thymidine kinase, and A-type inclusion body protein genes were identical to those of CPXV strains T2000 and E1989 previously identified in Finland (*3*). In phylogenetic analysis (Figure), CPXV/FIN/K2009 clustered with strains from Austria and shared ancestry with vaccinia virus. OPV IgG and IgM were detected by immunofluorescence assay (*3*) in serum samples up to 5 months after symptom onset (Technical Appendix Table 1).

**Figure:**
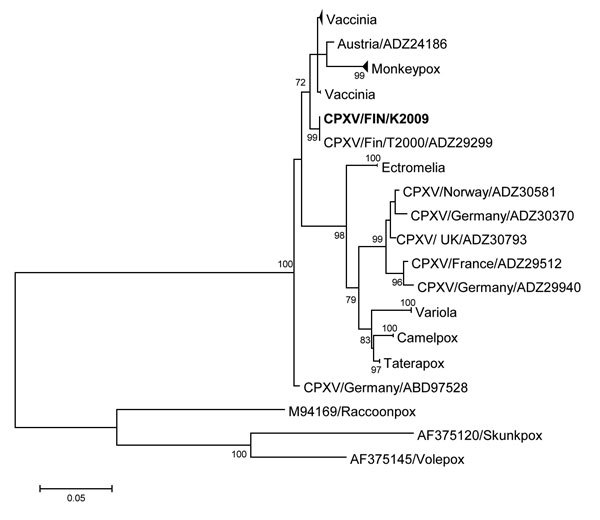
Phylogenetic tree of orthopoxviruses constructed on the basis of the hemagglutinin gene; boldface indicates the CPXV strain infecting the patient described in this article. The phylogeny shows that the sequence derived from this patient represents a locally circulating strain that shares ancestry with a few other CPXV strains and vaccinia virus. A maximum-likelihood tree was built with 1,000 bootstraps in MEGA 6.06 software (http://www.megasoftware.net/). MEGA was used to estimate the best nucleotide substitution model (general time reversible plus invariable sites). The sequence dataset was compiled from the Virus Pathogen Resource database (http://www.viprbrc.org) and aligned by using MUSCLE (http://www.ebi.ac.uk/Tools/msa/muscle/). Scale bar indicates nucleotide submissions per site. CPXV, cowpox virus.

The patient was started on intravenous polyclonal gammaglobulin and topical trifluorothymidine with in vitro anti-OPV effects; nevertheless, corneal erosions enlarged, corneal stromal edema ensued, and intraocular pressure increased (online Technical Appendix Figure, panel C), suggesting trabeculitis. Topical autologous serum drops had no effect. Periorbital edema slowly resolved, but corneal erosions persisted. Amniotic membrane transplantation (AMT) (*4*) was performed 2.5 months after onset. The inferior cornea melted, and the cornea lost transparency (online Technical Appendix Figure, panel D). AMT was repeated twice at 1-month intervals because of corneal thinning.

At 3.5 months after symptom onset, tecovirimat (400 mg 2×/d) was given orally for 14 days. Despite treatment, ocular OPV PCR test results remained positive until 9 months after onset (Technical Appendix Table 1), and corneal melting progressed (Technical Appendix Figure, panel E). Corneal collagen cross-linking and a fourth AMT were performed at 5 months after onset with partial success (Technical Appendix Figure, panel F).

At 1 year after symptom onset, corneal limbal stem cell deficiency with deep corneal neovascularization was evident. Autologous limbal stem cell transplantation from the patient’s other eye and another AMT were performed, resulting in stable corneal surface 2 months later (Technical Appendix Figure, panel G). Neovascularization regressed, the cornea cleared, and vision improved (Technical Appendix Figure, panels H, I).

Cowpox is transmitted to humans sporadically from rodents or cats (*5*). We snap-trapped 23 wild rodents from the yard of the patient’s home and from an adjacent meadow and trapped 136 rodents from 3 other regions 30–100 km from the patient’s home (Technical Appendix Table 2). We also collected 8 environmental samples from the patient’s storehouse. In accordance with the Finnish Act on Use of Animals for Experimental Purposes (62/2006) and the Finnish Animal Experiment Board’s later decision (May 16, 2007), the animal capture technique used is not an animal experiment and requires no ethics license. 

Diluted blood for IFA was collected from all rodents (*6*), and DNA was extracted from rodent liver and lungs and from environmental samples. One vole and 1 mouse from the meadow were seropositive for OPV; however, no CPXV DNA was amplifiable in the samples from the liver, lungs, or environment (online Technical Appendix Table 2). 

CPXV infection may manifest in severe ocular forms along with self-limiting cutaneous pocks (*5*). Our patient had keratitis with no other identifiable cause but CPXV. Culture and PCR from early conjunctival samples and serology confirmed the etiologic diagnosis.

Our case and that of another report (*7*) highlight the challenges of treating cowpox keratitis. Topical and systemic antiviral drugs and AMT appear ineffective during the acute phase. Corneal melting and scarring continued as long as CPXV was observed and until combined limbal stem cell and AMT treatment had favorable outcomes. Anamnesis of therapy-resistant keratitis should include information on rodent contacts.

We dated the infection to mid-August (incubation 7–21 days). Catching OPV-IgG–positive rodents close to the patient’s home 2 months after onset showed that OPVs were circulating in the local rodent population and indicated the putative role of CPXV-infected voles as the source of infection.

The latest cowpox outbreak in Central Europe involved several humans and pets (*8*). This patient was born in 1977, after Finland ceased smallpox vaccinations. Declining cross-reactive smallpox-vaccination immunity enables emergence of unusual cowpox infections in humans (*9*).

Technical Appendix. Diagnostic findings of patient, laboratory findings of sampling from rodents and environment, and images of progressive disease in eye of patient with ocular cowpox, Finland.
