# Supplementary material for: Severe Ocular Cowpox in a Human, Finland
Source: Emerg Infect Dis. 2015 Dec;21(12):2261–3. doi: 10.3201/eid2112.150621 (PMC4672437; doi:10.3201/eid2112.150621)
Supplement: Supplementary file 1 — Technical Appendix. Diagnostic findings of patient, laboratory findings of sampling from rodents and environment, and images of progressive disease in eye of patient with ocular cowpox, Finland. [file 15-0621-Techapp-s1.pdf]

Article DOI: http://dx.doi.org/10.3201/eid2112.150621

Severe Ocular Cowpox in a Human, Finland

Technical Appendix

Technical Appendix Table 1. Orthopoxvirus and cowpox virus diagnostic findings of the patient in Finland*

	Months after symptom onset, no.						
Analyte	0.5	1	3	4	5	8	≥9
OPV IgG titer	1,280	1,280	320	320	NA	NA	NA
OPV IgM titer	320	1,280	40	40	NA	NA	NA
Real-time PCR from conjunctival swab/ contact lens, HA gene, Cycle threshold values	+ Swab 33.7	NA	+ Swab 35.7	+ Swab 41.4/ Lens 43.1	+ Swab 35.4/ Lens 38.2	+ Swab 48.3	–
Virus isolation: CPE in Vero cells and electron microscopy	+ PCRs from cell culture + for genes HA, TK, ATIP, and 14 kDa protein	–	–	–	–	–	–

*OPV, Orthopoxvirus; NA, Not analyzed; CPE, Cytopathogenic effect; HA, Hemagglutinin, 1,091 bp, GenBank accession no. KR054112; TK, Thymidine kinase, 342 bp, KR054113; ATIP, A-type inclusion body protein, 1,590 bp, KR054111; +, positive; –, negative.

Technical Appendix Table 2. Results from the environment and rodents from the yard of the patient's home, its vicinity, and more distant regions

Sample description	No. samples	No. positive in IFA (%)	Comments on positive samples	No. positive in PCR* (HA and 14 kDa protein)
Environmental samples from the patient's outdoor storehouse†				
Dead insect, pieces of dry leaves, rubbish, dust, small pieces of paper	8	0		0
Rodents trapped in patient's yard and neighborhood				
Bank vole, *Myodes glareolus*	9	1 (11.1)	Male; antibodies had high avidity; mildly icteric.	0
Yellow-necked mouse, *Apodemus flavicollis*	14	1 (7.1)	Male; because of low titer, avidity was indeterminable.	0
Total rodents	23	2 (8.7)		0
Rodents trapped elsewhere in southern Finland (distance from patient's home)				
Mäntsälä (30 km)	33	7 (21.2)	Bank voles	NA
Riihimäki (50 km)	55	3 (5.5)	Bank voles, yellow-necked mouse	NA
Heinola (100 km)	48	41 (85.4)	Bank voles, field voles (*Microtus agrestis*)	NA

*HA, Hemagglutinin; NA, Not analyzed.
†Patient had cleaned the storehouse 1 week before onset.

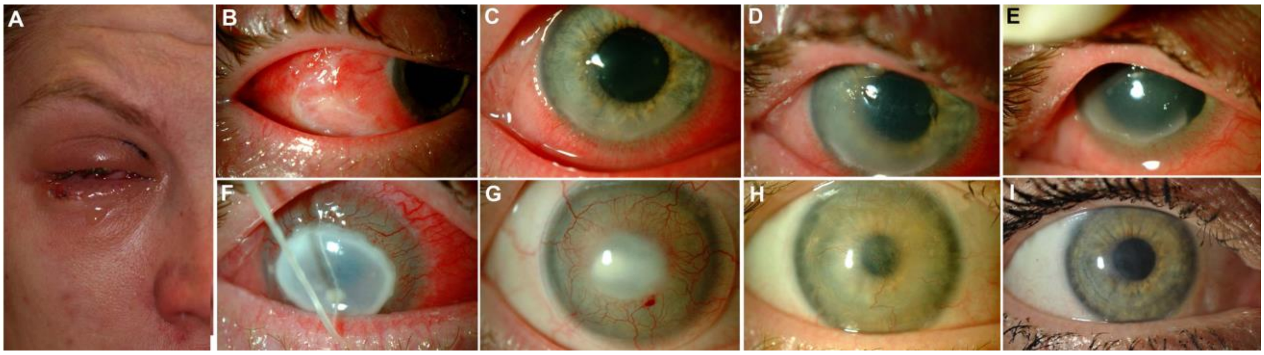

Technical Appendix Figure. Patient's facial image and a series of progressive cornea images after onset of disease. A) Facial image at 2 weeks. B) Cornea image at 2 weeks; C) at 2 months; D) at 3 months; E) at 5 months; F) at 7 months; G) at 15 months; H) at 30 months; I) at 4 years.
